# Two species of *Atteva* Walker, 1854 (Lepidoptera, Attevidae) new to Laos, with DNA barcodes

**DOI:** 10.3897/BDJ.14.e189183

**Published:** 2026-03-26

**Authors:** Sol-Moon Na, Na-Ri Shin, Yang-Seop Bae

**Affiliations:** 1 Animal and Plant Quarantine Agency, Gimcheon, Republic of Korea Animal and Plant Quarantine Agency Gimcheon Republic of Korea; 2 Incheon National University, Incheon, Republic of Korea Incheon National University Incheon Republic of Korea; 3 Incheon Green Environmental Center, Incheon, Republic of Korea Incheon Green Environmental Center Incheon Republic of Korea

**Keywords:** taxonomy, distribution, DNA barcoding, mitochondrial DNA

## Abstract

**Background:**

The tropical ermine moth genus *Atteva* Walker, 1854 is characterised by a vivid ground colour and an intricate pattern of white spots on the forewing, which often complicates species-level identification. Currently, only 52 species have been reported worldwide, with 20 species are known from the Oriental Region.

**New information:**

Two species, *A. niveigutta* Walker, 1854 and *A. sciodoxa* Meyrick, 1908, are newly recorded in Laos for the first time, supported by morphological characteristics and DNA barcodes.

## Introduction

The tropical ermine moth genus *Atteva* Walker, 1854 belongs to the family Attevidae and currently includes 52 described species worldwide, of which 20 species are known from the Oriental Region (Lewis and Sohn 2015, Na and Bae 2024).

The genus *Atteva* was established by Walker (1854) for the type species *Atteva niveigutta* Walker, 1854 from Bangladesh. To date, four species of *Atteva* have been reported from the Indochina Peninsula: *A. fabriciella* (Swederus, 1787), *A. niveigutta* Walker, 1854, *A. wallengreni* Sohn & Wu, 2013 and *A. chalco* Na & Bae, 2024. In Laos, only *A. fabriciella* (Swederus, 1787), has been reported (Kitajima 2020); however, that record includes a misspelling of the generic name (“Attiva”) and distributional information that requires confirmation.

Previous studies on *Atteva* have relied primarily on morphological characters and available COI barcode data are geographically biased towards the Australasian and Neotropical Regions. Although a small number of COI sequences of *Atteva* have been generated from the Oriental Region, publicly available data remain sparse and difficult to use for comparative analyses because of limited accompanying information. No COI barcode data have been reported from Laos.

In the present study, we provide illustrations of adults and male and female genitalia, diagnoses and re-descriptions of *A. niveigutta* Walker and *A. sciodoxa* Meyrick from Laos. In addition, we present the first available COI barcode data for *Atteva* from Laos, filling an important gap in the molecular taxonomy and biogeography of the genus.

## Materials and methods

### Specimen collection and preparation

The specimens were collected at the light traps with a mercury vapour lamp (220V/ 200W, Dongseong Co., Korea) and four ultraviolet lamps (20W, FL20SbL/18, Kumho Co., Korea). Genitalia were dissected and examined under a Leica EZ4 stereo-microscope (Leica Microsystems, GmbH, Wetzlar, Germany). Images of adults and genitalia were taken with a Leica M205C microscope (Leica Microsystems, Wetzlar, Germany) attached to a Dhyana 400DC CMOS camera (Tucsen Photonics, China). The examined specimens are deposited in the Plant Quarantine Technology Center, Animal and Plant Quarantine Agency, Gimcheon, Republic of Korea.

Abbreviations:

- PQTC: Plant Quarantine Technology Center, Animal and Plant Quarantine Agency;
- TL: type locality.

### DNA extraction and COI amplification and sequencing

Genomic DNA was extracted from adult specimens using the DNeasy Blood & Tissue Kit (QIAGEN, Hilden, Germany), following the manufacturer’s instructions. A fragment (~ 658 bp) of the mitochondrial cytochrome c oxidase subunit I (COI) gene was amplified by polymerase chain reaction (PCR) using the primer pair LCO1490 (5′-GGTCAACAAATCATCATAAAGATATTGG-3′) and HCO2198 (5′-TAAACTTCAGGGTGACCAAAAAATCA-3′) (Folmer et al. 1994). PCR amplification was performed under the following conditions: an initial denaturation at 95°C for 3 min, followed by 35 cycles of denaturation at 95°C for 30 s, annealing at 52°C for 30 s and extension at 72°C for 50 s, with a final extension at 72°C for 10 min. PCR reactions were carried out using AccuPower PCR Premix (Bioneer, Daejeon, South Korea). PCR products were verified by electrophoresis on 2% agarose E-Gels (Invitrogen, Carlsbad, CA, USA) and subsequently purified and sequenced bidirectionally using Sanger sequencing by BIONICS (Seoul, Republic of Korea). Raw sequence chromatograms were manually inspected and low-quality regions were trimmed prior to assembly. Forward and reverse sequences were assembled into consensus sequences for downstream analyses.

### DNA barcoding and Phylogenetic analysis

A total of 686 ingroup COI sequences of *Atteva* were analysed, including three newly-generated sequences from Laos (GenBank accession numbers: PX959790–PX959792). Additional sequences were downloaded from GenBank and the Barcode of Life Data Systems (BOLD; Ratnasingham and Hebert (2007); accessed 3 February 2026). For phylogenetic reconstruction, two outgroup sequences, *Plutella xylostella* (HM873752) and *Yponomeuta tokyonella* (KF523857), were included, resulting in a total dataset of 688 sequences. To reduce redundancy, identical COI haplotypes were collapsed within each species; however, identical haplotypes from different countries were retained to represent geographic coverage. Sequences were aligned using ClustalW (Thompson et al. 1994) implemented in MEGA version 11.0 (Tamura et al. 2021) and intra- and interspecific pairwise genetic distances were calculated under the Kimura 2-parameter (K2P) model (Kimura 1980). Phylogenetic relationships were inferred using the Neighbour-Joining (NJ) method (Saitou and Nei 1987) in MEGA version 11.0 and nodal support was assessed by bootstrap analysis with 1,000 replicates (Felsenstein 1985). For presentation, a representative subset of sequences was selected and shown in the NJ tree figure for clarity; the sequences included in the figure are listed in Table 1.

## Taxon treatments

### Atteva

Walker, 1854

4787AF94-5B68-54C0-AA93-C6F87EAAE16C

474D6C29-88DE-43F2-B9B6-6A5FCE72E3AF

Atteva Walker, 1854 - Walker (1854): 526. Types species: *Atteva niveigutta* Walker 1854, by monotypy.
Amblothridia Wallengern, 1861 - Wallengern (1861): 385. Type species: *Phalaena fabiriciella* Swederus, 1787, by subsequent designation by Fletcher (1929).
Carthara Walker, 1866 - *Walker (1866)*: 1871. Type species: *Carthara flavivitta* Walker, 1866b, by monotypy.
Corinea Walker, 1863 - *Walker (1863)*: 542. Type species: *Corinea niviguttella* Walker, 1863, by subsequent designation by Fletcher (1928).
Poeciloptera Clemens, 1861 - *Clemens (1861)*: 546. Type species: *Poeciloptera compta* Clemens, 1861, by monotypy.
Scintilla Guenée, 1879 - Guenée (1879): 287. Type species: *Tinea pastulella* Fabricius, 1787, by monotypy.
Syblis Guenée, 1879 - Guenée (1879): 288. Type species: *Syblis glaucopidella* Guenée, 1879, by monotypy.
Synadia Walker, 1866 - Walker (1866): 1984. Type species: *Carthara flavivitta* Walker, 1866 by monotypy.
Syncallia Guérin–Méneville, 1844 - *Guérin–Méneville (1844)*: 497. Type species: *Syncallia stellata* Guérin–Méneville, [1844] by monotypy.
Atteva niveigutta Walker, 1854List of the Specimens of Lepidopterous Insects in the Collection of the British Museum. Part II–Lepidoptera Heterocera: 526.

#### Diagnosis

Attevidae can be distinguished from other families of Yponomeutoidea by the following characters: (1) antennal scape without pecten; (2) maxillary palpi 3-segmented; (3) forewing lacking a pterostigma; (4) abdominal terga without spines; and (5) aedeagus without a basal scape and a W-shaped gnathos in the male genitalia (Dugdale et al. 1998).

### Atteva niveigutta

Walker, 1854

26196BC0-6688-5243-B295-072BD9CCD984

C48D2926-1BB6-484B-80E0-67E9ADC59171

Atteva niveigutta Walker, 1854 - *Walker (1854)*: 526. TL: Bangladesh (Sylhet).
Atteva niveiguttata Berg, 1880 - Berg (1880): 103. [misspelling of *A. niveigutta*].
Figs 1a, b, 2a, b, 3a.

#### Materials

- **Type status:** Other material. **Occurrence:** recordedBy: Bae Y.S., Na S.M., Lee D.J., KO J.H., Lee T.K.; individualCount: 2; sex: male; lifeStage: adult; establishmentMeans: Native; occurrenceStatus: Present; occurrenceID: 5012838D-2A1E-5829-BD4B-8AF0928056D1; **Taxon:** taxonID: Native; scientificNameID: urn:lsid:zoobank.org:act:CB5F8276-F223-4EB6-994C-809AB3CD5944; scientificName: Atteva niveigutta; parentNameUsage: Attevidae; namePublishedIn: Walker, Francis. 1854 List of the specimens of lepidopterous insects in the collection of the British Museum. Part II.—Lepidoptera Heterocera. British Museum, London. Vol. 2: 279-582.; phylum: Arthropoda; class: Insecta; order: Lepidoptera; family: Attevidae; genus: Atteva; specificEpithet: niveigutta; taxonRank: species; scientificNameAuthorship: Walker, 1854; vernacularName: Ailanthus webworms; taxonomicStatus: accepted; **Location:** country: Laos; countryCode: LA; stateProvince: Xieng Khouang Prov.; locality: Bantha; verbatimElevation: 1524 m; verbatimCoordinates: 19°44'50.20"N 103°37'28.10"E; georeferenceProtocol: label; **Identification:** identifiedBy: Sol-Moon Na; dateIdentified: 2025; **Event:** samplingProtocol: light traps; eventDate: 29-VI-2017; **Record Level:** language: en; collectionCode: Insects; basisOfRecord: PreservedSpecimen
- **Type status:** Other material. **Occurrence:** recordedBy: Bae Y.S., Na S.M., Lee D.J., KO J.H., Lee T.K.; individualCount: 1; sex: female; lifeStage: adult; establishmentMeans: Native; occurrenceStatus: Present; occurrenceID: 9F08D023-75C0-518A-B9A6-11EDDAB3AF4B; **Taxon:** taxonID: Native; scientificNameID: urn:lsid:zoobank.org:act:CB5F8276-F223-4EB6-994C-809AB3CD5944; scientificName: Atteva niveigutta; parentNameUsage: Attevidae; namePublishedIn: Walker, Francis. 1854 List of the specimens of lepidopterous insects in the collection of the British Museum. Part II.—Lepidoptera Heterocera. British Museum, London. Vol. 2: 279-582.; phylum: Arthropoda; class: Insecta; order: Lepidoptera; family: Attevidae; genus: Atteva; specificEpithet: niveigutta; taxonRank: species; scientificNameAuthorship: Walker, 1854; vernacularName: Ailanthus webworms; taxonomicStatus: accepted; **Location:** country: Laos; countryCode: LA; stateProvince: Xieng Khouang Prov.; locality: Bantha; verbatimElevation: 1524 m; verbatimCoordinates: 19°44'50.20"N 103°37'28.10"E; georeferenceProtocol: label; **Identification:** identifiedBy: Sol-Moon Na; dateIdentified: 2025; **Event:** samplingProtocol: light traps; eventDate: 29-VI-2017; **Record Level:** language: en; collectionCode: Insects; basisOfRecord: PreservedSpecimen
- **Type status:** Other material. **Occurrence:** recordedBy: Bae Y.S., Na S.M., Lee D.J., KO J.H., Lee T.K.; individualCount: 6; sex: male; lifeStage: adult; establishmentMeans: Native; occurrenceStatus: Present; occurrenceID: ADD3EA98-26E0-5A0C-95D1-B9FF7FC01FEB; **Taxon:** taxonID: Native; scientificNameID: urn:lsid:zoobank.org:act:CB5F8276-F223-4EB6-994C-809AB3CD5944; scientificName: Atteva niveigutta; parentNameUsage: Attevidae; namePublishedIn: Walker, Francis. 1854 List of the specimens of lepidopterous insects in the collection of the British Museum. Part II.—Lepidoptera Heterocera. British Museum, London. Vol. 2: 279-582.; kingdom: Anmalia; phylum: Arthropoda; class: Insecta; order: Lepidoptera; family: Attevidae; genus: Atteva; specificEpithet: niveigutta; taxonRank: species; scientificNameAuthorship: Walker, 1854; vernacularName: Ailanthus webworms; taxonomicStatus: accepted; **Location:** country: Laos; countryCode: LA; stateProvince: Xieng Khouang Prov.; locality: Bantha; verbatimElevation: 1524 m; verbatimCoordinates: 19°44'50.20"N 103°37'28.10"E; georeferenceProtocol: label; **Identification:** identifiedBy: Sol-Moon Na; dateIdentified: 2025; **Event:** samplingProtocol: light traps; eventDate: 1-VII-2017; **Record Level:** language: en; collectionCode: Insects; basisOfRecord: PreservedSpecimen
- **Type status:** Other material. **Occurrence:** recordedBy: Bae Y.S., Na S.M., Lee D.J., KO J.H., Lee T.K.; individualCount: 1; sex: female; lifeStage: adult; establishmentMeans: Native; occurrenceStatus: Present; occurrenceID: E2451EC3-8ADF-5819-B9C2-890279EDA70F; **Taxon:** taxonID: Native; scientificNameID: urn:lsid:zoobank.org:act:CB5F8276-F223-4EB6-994C-809AB3CD5944; scientificName: Atteva niveigutta; parentNameUsage: Attevidae; namePublishedIn: Walker, Francis. 1854 List of the specimens of lepidopterous insects in the collection of the British Museum. Part II.—Lepidoptera Heterocera. British Museum, London. Vol. 2: 279-582.; phylum: Arthropoda; class: Insecta; order: Lepidoptera; family: Attevidae; genus: Atteva; specificEpithet: niveigutta; taxonRank: species; scientificNameAuthorship: Walker, 1854; vernacularName: Ailanthus webworms; taxonomicStatus: accepted; **Location:** country: Laos; countryCode: LA; stateProvince: Xieng Khouang Prov.; locality: Bantha; verbatimElevation: 1524 m; verbatimCoordinates: 19°44'50.20"N 103°37'28.10"E; georeferenceProtocol: label; **Identification:** identifiedBy: Sol-Moon Na; dateIdentified: 2025; **Event:** samplingProtocol: light traps; eventDate: 1-VII-2017; **Record Level:** language: en; collectionCode: Insects; basisOfRecord: PreservedSpecimen
- **Type status:** Other material. **Occurrence:** recordedBy: Bae Y.S., Park B.S., Na S.M., Kim, J.W., Lee D.J.; individualCount: 1; sex: female; lifeStage: adult; establishmentMeans: Native; occurrenceStatus: Present; occurrenceID: DF49D539-5F30-584B-8032-FB68D78786C7; **Taxon:** taxonID: Native; scientificName: Atteva sciodoxa; parentNameUsage: Attevidae; namePublishedIn: Meyrick, 1908 XXXIX—New Micro-Lepidoptera from India and Burma. Records of the Zoological Survey of India 2(4), 395–400.; phylum: Arthropoda; class: Insecta; order: Lepidoptera; family: Attevidae; genus: Atteva; specificEpithet: sciodoxa; taxonRank: species; scientificNameAuthorship: Meyrick, 1908; vernacularName: Tongkat Ali Tiger Moth; taxonomicStatus: accepted; **Location:** country: Laos; countryCode: LA; stateProvince: Bolikhamsai Prov.; locality: Tad Xai Waterfall; verbatimElevation: 321 m; verbatimCoordinates: 18°27'05.98"N 103°8'40.06"E; georeferenceProtocol: label; **Identification:** identifiedBy: Sol-Moon Na; dateIdentified: 2025; **Event:** samplingProtocol: light traps; eventDate: 8-XI-2015; **Record Level:** language: en; collectionCode: Insects; basisOfRecord: PreservedSpecimen
- **Type status:** Other material. **Occurrence:** recordedBy: Bae Y.S., Park B.S., Na S.M., Kim, J.W., Lee D.J.; individualCount: 1; sex: male; lifeStage: adult; establishmentMeans: Native; occurrenceStatus: Present; occurrenceID: CB99B2BB-A88D-5CE3-AA7F-25294DE7F438; **Taxon:** taxonID: Native; scientificName: Atteva sciodoxa; parentNameUsage: Attevidae; namePublishedIn: Meyrick, 1908 XXXIX—New Micro-Lepidoptera from India and Burma. Records of the Zoological Survey of India 2(4), 395–400.; phylum: Arthropoda; class: Insecta; order: Lepidoptera; family: Attevidae; genus: Atteva; specificEpithet: sciodoxa; taxonRank: species; scientificNameAuthorship: Meyrick, 1908; vernacularName: Tongkat Ali Tiger Moth; taxonomicStatus: accepted; **Location:** country: Laos; countryCode: LA; stateProvince: Xieng Khouang Prov.; locality: Bantha; verbatimElevation: 1298 m; verbatimCoordinates: 19°45'07.35"N 103°33'25.34"E; georeferenceProtocol: label; **Identification:** identifiedBy: Sol-Moon Na; dateIdentified: 2025; **Event:** samplingProtocol: light traps; eventDate: 4-IV-2016; **Record Level:** language: en; collectionCode: Insects; basisOfRecord: PreservedSpecimen
- **Type status:** Other material. **Occurrence:** recordedBy: Bae Y.S., Park B.S., Na S.M., Kim, J.W., Lee D.J.; individualCount: 1; sex: female; lifeStage: adult; establishmentMeans: Native; occurrenceStatus: Present; occurrenceID: 3E4A023F-58F5-5701-8092-D4E822556987; **Taxon:** taxonID: Native; scientificName: Atteva sciodoxa; parentNameUsage: Attevidae; namePublishedIn: Meyrick, 1908 XXXIX—New Micro-Lepidoptera from India and Burma. Records of the Zoological Survey of India 2(4), 395–400.; phylum: Arthropoda; class: Insecta; order: Lepidoptera; family: Attevidae; genus: Atteva; specificEpithet: sciodoxa; taxonRank: species; scientificNameAuthorship: Meyrick, 1908; vernacularName: Tongkat Ali Tiger Moth; taxonomicStatus: accepted; **Location:** country: Laos; countryCode: LA; stateProvince: Xieng Khouang Prov.; locality: Bantha; verbatimElevation: 1298 m; verbatimCoordinates: 19°45'07.35"N 103°33'25.34"E; georeferenceProtocol: label; **Identification:** identifiedBy: Sol-Moon Na; dateIdentified: 2025; **Event:** samplingProtocol: light traps; eventDate: 4-IV-2016; **Record Level:** language: en; collectionCode: Insects; basisOfRecord: PreservedSpecimen
- **Type status:** Other material. **Occurrence:** recordedBy: Bae Y.S., Park B.S., Na S.M., Kim, J.W., Lee D.J.; individualCount: 3; sex: female; lifeStage: adult; establishmentMeans: Native; occurrenceStatus: Present; occurrenceID: C5B33836-0C43-521C-AFE3-2D1950B43EDF; **Taxon:** taxonID: Native; scientificName: Atteva sciodoxa; parentNameUsage: Attevidae; namePublishedIn: Meyrick, 1908 XXXIX—New Micro-Lepidoptera from India and Burma. Records of the Zoological Survey of India 2(4), 395–400.; phylum: Arthropoda; class: Insecta; order: Lepidoptera; family: Attevidae; genus: Atteva; specificEpithet: sciodoxa; taxonRank: species; scientificNameAuthorship: Meyrick, 1908; vernacularName: Tongkat Ali Tiger Moth; taxonomicStatus: accepted; **Location:** country: Laos; countryCode: LA; stateProvince: Xieng Khouang Prov.; locality: Bantha; verbatimElevation: 1298 m; verbatimCoordinates: 19°45'07.35"N 103°33'25.34"E; georeferenceProtocol: label; **Identification:** identifiedBy: Sol-Moon Na; dateIdentified: 2025; **Event:** samplingProtocol: light traps; eventDate: 6-IV-2016; **Record Level:** language: en; collectionCode: Insects; basisOfRecord: PreservedSpecimen
- **Type status:** Other material. **Occurrence:** recordedBy: Bae Y.S., Park B.S., Na S.M., Kim, J.W., Lee D.J.; individualCount: 1; sex: female; lifeStage: adult; establishmentMeans: Native; occurrenceStatus: Present; occurrenceID: 92142DC8-1841-5C2B-874B-B653F4A157F5; **Taxon:** taxonID: Native; scientificName: Atteva sciodoxa; parentNameUsage: Attevidae; namePublishedIn: Meyrick, 1908 XXXIX—New Micro-Lepidoptera from India and Burma. Records of the Zoological Survey of India 2(4), 395–400.; phylum: Arthropoda; class: Insecta; order: Lepidoptera; family: Attevidae; genus: Atteva; specificEpithet: sciodoxa; taxonRank: species; scientificNameAuthorship: Meyrick, 1908; vernacularName: Tongkat Ali Tiger Moth; taxonomicStatus: accepted; **Location:** country: Laos; countryCode: LA; stateProvince: Xieng Khouang Prov.; locality: Bantha; verbatimElevation: 1298 m; verbatimCoordinates: 19°45'07.35"N 103°33'25.34"E; georeferenceProtocol: label; **Identification:** identifiedBy: Sol-Moon Na; dateIdentified: 2025; **Event:** samplingProtocol: light traps; eventDate: 6-IV-2016; **Record Level:** language: en; collectionCode: Insects; basisOfRecord: PreservedSpecimen
- **Type status:** Other material. **Occurrence:** recordedBy: Bae Y.S., Park B.S., Na S.M., Lee D.J., Ko J.H.; individualCount: 1; sex: female; lifeStage: adult; establishmentMeans: Native; occurrenceStatus: Present; occurrenceID: 42B898EE-CB42-50B4-A13D-0E4686FC07FF; **Taxon:** taxonID: Native; scientificName: Atteva sciodoxa; parentNameUsage: Attevidae; namePublishedIn: Meyrick, 1908 XXXIX—New Micro-Lepidoptera from India and Burma. Records of the Zoological Survey of India 2(4), 395–400.; phylum: Arthropoda; class: Insecta; order: Lepidoptera; family: Attevidae; genus: Atteva; specificEpithet: sciodoxa; taxonRank: species; scientificNameAuthorship: Meyrick, 1908; vernacularName: Tongkat Ali Tiger Moth; taxonomicStatus: accepted; **Location:** country: Laos; countryCode: LA; stateProvince: Bolikhamsai Prov.; locality: PKK Natl` Park; verbatimElevation: 624 m; verbatimCoordinates: 18°30'12.20"N 103°3'40.50"E; georeferenceProtocol: label; **Identification:** identifiedBy: Sol-Moon Na; dateIdentified: 2025; **Event:** samplingProtocol: light traps; eventDate: 18-II-2017; **Record Level:** language: en; collectionCode: Insects; basisOfRecord: PreservedSpecimen
- **Type status:** Other material. **Occurrence:** recordedBy: Bae Y.S., Na S.M., Lee D.J., Ko J.H. Lee T.K., Cha Y.B., Jang C.M.; individualCount: 1; sex: female; lifeStage: adult; establishmentMeans: Native; occurrenceStatus: Present; occurrenceID: 214A5A74-BCC8-5F07-B618-C4DABD8C8CBC; **Taxon:** taxonID: Native; scientificName: Atteva sciodoxa; parentNameUsage: Attevidae; namePublishedIn: Meyrick, 1908 XXXIX—New Micro-Lepidoptera from India and Burma. Records of the Zoological Survey of India 2(4), 395–400.; phylum: Arthropoda; class: Insecta; order: Lepidoptera; family: Attevidae; genus: Atteva; specificEpithet: sciodoxa; taxonRank: species; scientificNameAuthorship: Meyrick, 1908; vernacularName: Tongkat Ali Tiger Moth; taxonomicStatus: accepted; **Location:** country: Laos; countryCode: LA; stateProvince: Bolikhamsai Prov.; locality: PKK Natl` Park; verbatimElevation: 290 m; verbatimCoordinates: 18°25'11.75"N 103°5'12.59"E; georeferenceProtocol: label; **Identification:** identifiedBy: Sol-Moon Na; dateIdentified: 2025; **Event:** samplingProtocol: light traps; eventDate: 3-IX-2018; **Record Level:** language: en; collectionCode: Insects; basisOfRecord: PreservedSpecimen
- **Type status:** Other material. **Occurrence:** recordedBy: Bae Y.S., Qi M.J., Lee D.J., Ko J.H., Lee T.K., Cha Y.B., Jang C.M.; individualCount: 1; sex: female; lifeStage: adult; establishmentMeans: Native; occurrenceStatus: Present; occurrenceID: 0CDC8358-60D0-57FA-8171-32A19468AC61; **Taxon:** taxonID: Native; scientificName: Atteva sciodoxa; parentNameUsage: Attevidae; namePublishedIn: Meyrick, 1908 XXXIX—New Micro-Lepidoptera from India and Burma. Records of the Zoological Survey of India 2(4), 395–400.; phylum: Arthropoda; class: Insecta; order: Lepidoptera; family: Attevidae; genus: Atteva; specificEpithet: sciodoxa; taxonRank: species; scientificNameAuthorship: Meyrick, 1908; vernacularName: Tongkat Ali Tiger Moth; taxonomicStatus: accepted; **Location:** country: Laos; countryCode: LA; stateProvince: Bolikhamsai Prov.; locality: PKK Natl` Park; verbatimElevation: 470 m; verbatimCoordinates: 18°27'23.76"N 103°5'5.15"E; georeferenceProtocol: label; **Identification:** identifiedBy: Sol-Moon Na; dateIdentified: 2025; **Event:** samplingProtocol: light traps; eventDate: 26-VII-2019; **Record Level:** language: en; collectionCode: Insects; basisOfRecord: PreservedSpecimen

#### Description

Adult (Fig. 1a, b). Wingspan 26–31 mm (n = 8). Vertex tuft with dark grey scales, frons with appressed white scales continuing to compound eye and dark grey patch at middle. Antenna filiform, 3/5 length of the forewing, scape white, flagellum posterior half dark brown and anterior half white. Labial palpus dark grey, slender, upcurved, pointed at tip, scattered with white scales inner margin. Patagium orange-brown, white transverse scales 1/4 anteriorly; tegula orange-brown, with white marking near wing base; mesoscutum orange-brown, with two white dots at 2/3 posteriorly. Fore-legs with coxa white, femur orange-brown on outer margin, white on inner margin, tibia pale brown outer margin, white inner margin, 1^st^ segment of tarsus pale brown with three white spots at both ends and middle, the other tarsus pale brown; mid-leg with coxa white, femur orange-brown on outer margin, white on inner margin, femur outer margin pale brown, inner margin white elongated to middle of outer margin and posterior end, tibia outer margin pale brown, inner margin white elongated to middle of outer margin and white at posterior end and spur, tarsus pale brown; hind-leg pale orange with bristles at tibia. Fore-wing base colour orange brown or pale brown with 35–49 white dots and fringe same as base colour. Hind-wing and fringe orange or orange-brown. Abdomen orange or orange-brown.

Male genitalia (Fig. 2a, b): Uncus rectangular, bifid, each process half length of socius; socius apically elongated triangular, long hairs from base to basal 3/4, pointed apex with a comb-like spinose zone near to apex; gnathal process slender, starting from broad arm and curve upwardly, sclerotised, swollen at apex, 1/2 as long as saccus. Valva elongated elliptical, convex from basal 1/3 to apex, elongated at tip, about 3 times as long as socius; sacculus lobate at base. Saccus slender, about 1/2 length of valva. Aedeagus stout, slightly curved at middle, about 1.5 times as long as valva; with cornuti composed of four rows of spinules, from apex to posterior half.

Female genitalia (Fig. 3a): Papilla analis bifid, semi-oval with hairs. Apophyses posteriores as long as apophyses anteriores. Lamella postvaginalis shallow, setose, semi-circular lobes. Ostium rounded. Ductus bursae slender, 1/2 times as long as corpus bursae; antrum sclerotised, at anterior half of ductus bursae, enlarged to corpus bursae. Corpus bursae ovate, membranous; signum elliptical plate, narrow to apex, with exterior denticules dense.

#### Diagnosis

This species is superficially similar to *A. yanguifella* Sohn & Wu, but it can be distinguished by the following genital characters: posterior processes of uncus are broader than saccus, apex of medial gnathal plate is swollen, the saccus is half-length of valva, the cornuti have two large spines and one small spine; antrum is enlarged near the corpus bursae, signum is elliptical, weakly sclerotised medial part of signum broad (Sohn and Wu 2013).

#### Distribution

Laos (present study), Bangladesh, China (uncertain), India (Assam), Nepal, Taiwan (introduced?), Thailand (Sohn and Wu 2013, Lewis and Sohn 2015).

#### Ecology

**Host plant.** *Ailanthus excelsus* Roxb. (Simaroubaceae) (Moore 1867).

#### Notes

This species is reported from Laos for the first time.

### Atteva sciodoxa

Meyrick, 1908

36266025-8050-5FDE-90A1-04B201C5209F

Atteva sciodoxa Meyrick, 1908 - *Meyrick (1908)* : 398. TL: Malaysia, Myanmar.

#### Materials

- **Type status:** Other material. **Occurrence:** recordedBy: Bae Y.S., Na S.M., Lee D.J., KO J.H., Lee T.K.; individualCount: 2; sex: male; lifeStage: adult; establishmentMeans: Native; occurrenceStatus: Present; occurrenceID: 505F5FA5-11A5-5556-B5C2-7B4BD2F79BDE; **Taxon:** taxonID: Native; scientificNameID: urn:lsid:zoobank.org:act:CB5F8276-F223-4EB6-994C-809AB3CD5944; scientificName: Atteva niveigutta; parentNameUsage: Attevidae; namePublishedIn: Walker, Francis. 1854 List of the specimens of lepidopterous insects in the collection of the British Museum. Part II.—Lepidoptera Heterocera. British Museum, London. Vol. 2: 279-582.; phylum: Arthropoda; class: Insecta; order: Lepidoptera; family: Attevidae; genus: Atteva; specificEpithet: niveigutta; taxonRank: species; scientificNameAuthorship: Walker, 1854; vernacularName: Ailanthus webworms; taxonomicStatus: accepted; **Location:** country: Laos; countryCode: LA; stateProvince: Xieng Khouang Prov.; locality: Bantha; verbatimElevation: 1524 m; verbatimCoordinates: 19°44'50.20"N 103°37'28.10"E; georeferenceProtocol: label; **Identification:** identifiedBy: Sol-Moon Na; dateIdentified: 2025; **Event:** samplingProtocol: light traps; eventDate: 29-VI-2017; **Record Level:** language: en; collectionCode: Insects; basisOfRecord: PreservedSpecimen
- **Type status:** Other material. **Occurrence:** recordedBy: Bae Y.S., Na S.M., Lee D.J., KO J.H., Lee T.K.; individualCount: 1; sex: female; lifeStage: adult; establishmentMeans: Native; occurrenceStatus: Present; occurrenceID: BF365902-B613-590C-90DE-5AD9FEF1668D; **Taxon:** taxonID: Native; scientificNameID: urn:lsid:zoobank.org:act:CB5F8276-F223-4EB6-994C-809AB3CD5944; scientificName: Atteva niveigutta; parentNameUsage: Attevidae; namePublishedIn: Walker, Francis. 1854 List of the specimens of lepidopterous insects in the collection of the British Museum. Part II.—Lepidoptera Heterocera. British Museum, London. Vol. 2: 279-582.; phylum: Arthropoda; class: Insecta; order: Lepidoptera; family: Attevidae; genus: Atteva; specificEpithet: niveigutta; taxonRank: species; scientificNameAuthorship: Walker, 1854; vernacularName: Ailanthus webworms; taxonomicStatus: accepted; **Location:** country: Laos; countryCode: LA; stateProvince: Xieng Khouang Prov.; locality: Bantha; verbatimElevation: 1524 m; verbatimCoordinates: 19°44'50.20"N 103°37'28.10"E; georeferenceProtocol: label; **Identification:** identifiedBy: Sol-Moon Na; dateIdentified: 2025; **Event:** samplingProtocol: light traps; eventDate: 29-VI-2017; **Record Level:** language: en; collectionCode: Insects; basisOfRecord: PreservedSpecimen
- **Type status:** Other material. **Occurrence:** recordedBy: Bae Y.S., Na S.M., Lee D.J., KO J.H., Lee T.K.; individualCount: 6; sex: male; lifeStage: adult; establishmentMeans: Native; occurrenceStatus: Present; occurrenceID: 7E906247-A872-5D7B-AAF1-1FFAFA0093A3; **Taxon:** taxonID: Native; scientificNameID: urn:lsid:zoobank.org:act:CB5F8276-F223-4EB6-994C-809AB3CD5944; scientificName: Atteva niveigutta; parentNameUsage: Attevidae; namePublishedIn: Walker, Francis. 1854 List of the specimens of lepidopterous insects in the collection of the British Museum. Part II.—Lepidoptera Heterocera. British Museum, London. Vol. 2: 279-582.; phylum: Arthropoda; class: Insecta; order: Lepidoptera; family: Attevidae; genus: Atteva; specificEpithet: niveigutta; taxonRank: species; scientificNameAuthorship: Walker, 1854; vernacularName: Ailanthus webworms; taxonomicStatus: accepted; **Location:** country: Laos; countryCode: LA; stateProvince: Xieng Khouang Prov.; locality: Bantha; verbatimElevation: 1524 m; verbatimCoordinates: 19°44'50.20"N 103°37'28.10"E; georeferenceProtocol: label; **Identification:** identifiedBy: Sol-Moon Na; dateIdentified: 2025; **Event:** samplingProtocol: light traps; eventDate: 1-VII-2017; **Record Level:** language: en; collectionCode: Insects; basisOfRecord: PreservedSpecimen
- **Type status:** Other material. **Occurrence:** recordedBy: Bae Y.S., Na S.M., Lee D.J., KO J.H., Lee T.K.; individualCount: 1; sex: female; lifeStage: adult; establishmentMeans: Native; occurrenceStatus: Present; occurrenceID: 98C12276-7A17-5059-AE6E-2BB0F173BD7A; **Taxon:** taxonID: Native; scientificNameID: urn:lsid:zoobank.org:act:CB5F8276-F223-4EB6-994C-809AB3CD5944; scientificName: Atteva niveigutta; parentNameUsage: Attevidae; namePublishedIn: Walker, Francis. 1854 List of the specimens of lepidopterous insects in the collection of the British Museum. Part II.—Lepidoptera Heterocera. British Museum, London. Vol. 2: 279-582.; phylum: Arthropoda; class: Insecta; order: Lepidoptera; family: Attevidae; genus: Atteva; specificEpithet: niveigutta; taxonRank: species; scientificNameAuthorship: Walker, 1854; vernacularName: Ailanthus webworms; taxonomicStatus: accepted; **Location:** country: Laos; countryCode: LA; stateProvince: Xieng Khouang Prov.; locality: Bantha; verbatimElevation: 1524 m; verbatimCoordinates: 19°44'50.20"N 103°37'28.10"E; georeferenceProtocol: label; **Identification:** identifiedBy: Sol-Moon Na; dateIdentified: 2025; **Event:** samplingProtocol: light traps; eventDate: 1-VII-2017; **Record Level:** language: en; collectionCode: Insects; basisOfRecord: PreservedSpecimen
- **Type status:** Other material. **Occurrence:** recordedBy: Bae Y.S., Park B.S., Na S.M., Kim, J.W., Lee D.J.; individualCount: 1; sex: female; lifeStage: adult; establishmentMeans: Native; occurrenceStatus: Present; occurrenceID: 2CE8C3DC-0619-56B0-A455-F6D1BDD3AFA8; **Taxon:** taxonID: Native; scientificName: Atteva sciodoxa; parentNameUsage: Attevidae; namePublishedIn: Meyrick, 1908 XXXIX—New Micro-Lepidoptera from India and Burma. Records of the Zoological Survey of India 2(4), 395–400.; phylum: Arthropoda; class: Insecta; order: Lepidoptera; family: Attevidae; genus: Atteva; specificEpithet: sciodoxa; taxonRank: species; scientificNameAuthorship: Meyrick, 1908; vernacularName: Tongkat Ali Tiger Moth; taxonomicStatus: accepted; **Location:** country: Laos; countryCode: LA; stateProvince: Bolikhamsai Prov.; locality: Tad Xai Waterfall; verbatimElevation: 321 m; verbatimCoordinates: 18°27'05.98"N 103°8'40.06"E; georeferenceProtocol: label; **Identification:** identifiedBy: Sol-Moon Na; dateIdentified: 2025; **Event:** samplingProtocol: light traps; eventDate: 8-XI-2015; **Record Level:** language: en; collectionCode: Insects; basisOfRecord: PreservedSpecimen
- **Type status:** Other material. **Occurrence:** recordedBy: Bae Y.S., Park B.S., Na S.M., Kim, J.W., Lee D.J.; individualCount: 1; sex: male; lifeStage: adult; establishmentMeans: Native; occurrenceStatus: Present; occurrenceID: 645473BD-737C-576C-823B-EEFECA1BCCD3; **Taxon:** taxonID: Native; scientificName: Atteva sciodoxa; parentNameUsage: Attevidae; namePublishedIn: Meyrick, 1908 XXXIX—New Micro-Lepidoptera from India and Burma. Records of the Zoological Survey of India 2(4), 395–400.; phylum: Arthropoda; class: Insecta; order: Lepidoptera; family: Attevidae; genus: Atteva; specificEpithet: sciodoxa; taxonRank: species; scientificNameAuthorship: Meyrick, 1908; vernacularName: Tongkat Ali Tiger Moth; taxonomicStatus: accepted; **Location:** country: Laos; countryCode: LA; stateProvince: Xieng Khouang Prov.; locality: Bantha; verbatimElevation: 1298 m; verbatimCoordinates: 19°45'07.35"N 103°33'25.34"E; georeferenceProtocol: label; **Identification:** identifiedBy: Sol-Moon Na; dateIdentified: 2025; **Event:** samplingProtocol: light traps; eventDate: 2016-IV-4; **Record Level:** language: en; collectionCode: Insects; basisOfRecord: PreservedSpecimen
- **Type status:** Other material. **Occurrence:** recordedBy: Bae Y.S., Park B.S., Na S.M., Kim, J.W., Lee D.J.; individualCount: 1; sex: female; lifeStage: adult; establishmentMeans: Native; occurrenceStatus: Present; occurrenceID: 8FE2AA7B-F543-5E55-99CE-AAAB16D805A7; **Taxon:** taxonID: Native; scientificName: Atteva sciodoxa; parentNameUsage: Attevidae; namePublishedIn: Meyrick, 1908 XXXIX—New Micro-Lepidoptera from India and Burma. Records of the Zoological Survey of India 2(4), 395–400.; phylum: Arthropoda; class: Insecta; order: Lepidoptera; family: Attevidae; genus: Atteva; specificEpithet: sciodoxa; taxonRank: species; scientificNameAuthorship: Meyrick, 1908; vernacularName: Tongkat Ali Tiger Moth; taxonomicStatus: accepted; **Location:** country: Laos; countryCode: LA; stateProvince: Xieng Khouang Prov.; locality: Bantha; verbatimElevation: 1298 m; verbatimCoordinates: 19°45'07.35"N 103°33'25.34"E; georeferenceProtocol: label; **Identification:** identifiedBy: Sol-Moon Na; dateIdentified: 2025; **Event:** samplingProtocol: light traps; eventDate: 4-IV-2016; **Record Level:** language: en; collectionCode: Insects; basisOfRecord: PreservedSpecimen
- **Type status:** Other material. **Occurrence:** recordedBy: Bae Y.S., Park B.S., Na S.M., Kim, J.W., Lee D.J.; individualCount: 3; sex: female; lifeStage: adult; establishmentMeans: Native; occurrenceStatus: Present; occurrenceID: 726E5F82-3D0E-58DD-8C54-D4B0E0FC700D; **Taxon:** taxonID: Native; scientificName: Atteva sciodoxa; parentNameUsage: Attevidae; namePublishedIn: Meyrick, 1908 XXXIX—New Micro-Lepidoptera from India and Burma. Records of the Zoological Survey of India 2(4), 395–400.; phylum: Arthropoda; class: Insecta; order: Lepidoptera; family: Attevidae; genus: Atteva; specificEpithet: sciodoxa; taxonRank: species; scientificNameAuthorship: Meyrick, 1908; vernacularName: Tongkat Ali Tiger Moth; taxonomicStatus: accepted; **Location:** country: Laos; countryCode: LA; stateProvince: Xieng Khouang Prov.; locality: Bantha; verbatimElevation: 1298 m; verbatimCoordinates: 19°45'07.35"N 103°33'25.34"E; georeferenceProtocol: label; **Identification:** identifiedBy: Sol-Moon Na; dateIdentified: 2025; **Event:** samplingProtocol: light traps; eventDate: 6-IV-2016; **Record Level:** language: en; collectionCode: Insects; basisOfRecord: PreservedSpecimen
- **Type status:** Other material. **Occurrence:** recordedBy: Bae Y.S., Park B.S., Na S.M., Kim, J.W., Lee D.J.; individualCount: 1; sex: female; lifeStage: adult; establishmentMeans: Native; occurrenceStatus: Present; occurrenceID: 964F7A89-7BEE-585D-B014-490B65DB3A78; **Taxon:** taxonID: Native; scientificName: Atteva sciodoxa; parentNameUsage: Attevidae; namePublishedIn: Meyrick, 1908 XXXIX—New Micro-Lepidoptera from India and Burma. Records of the Zoological Survey of India 2(4), 395–400.; phylum: Arthropoda; class: Insecta; order: Lepidoptera; family: Attevidae; genus: Atteva; specificEpithet: sciodoxa; taxonRank: species; scientificNameAuthorship: Meyrick, 1908; vernacularName: Tongkat Ali Tiger Moth; taxonomicStatus: accepted; **Location:** country: Laos; countryCode: LA; stateProvince: Xieng Khouang Prov.; locality: Bantha; verbatimElevation: 1298 m; verbatimCoordinates: 19°45'07.35"N 103°33'25.34"E; georeferenceProtocol: label; **Identification:** identifiedBy: Sol-Moon Na; dateIdentified: 2025; **Event:** samplingProtocol: light traps; eventDate: 6-IV-2016; **Record Level:** language: en; collectionCode: Insects; basisOfRecord: PreservedSpecimen
- **Type status:** Other material. **Occurrence:** recordedBy: Bae Y.S., Park B.S., Na S.M., Lee D.J., Ko J.H.; individualCount: 1; sex: female; lifeStage: adult; establishmentMeans: Native; occurrenceStatus: Present; occurrenceID: C368A68F-F43B-59AA-A26F-2DC72A5165BB; **Taxon:** taxonID: Native; scientificName: Atteva sciodoxa; parentNameUsage: Attevidae; namePublishedIn: Meyrick, 1908 XXXIX—New Micro-Lepidoptera from India and Burma. Records of the Zoological Survey of India 2(4), 395–400.; phylum: Arthropoda; class: Insecta; order: Lepidoptera; family: Attevidae; genus: Atteva; specificEpithet: sciodoxa; taxonRank: species; scientificNameAuthorship: Meyrick, 1908; vernacularName: Tongkat Ali Tiger Moth; taxonomicStatus: accepted; **Location:** country: Laos; countryCode: LA; stateProvince: Bolikhamsai Prov.; locality: PKK Natl` Park; verbatimElevation: 624 m; verbatimCoordinates: 18°30'12.20"N 103°3'40.50"E; georeferenceProtocol: label; **Identification:** identifiedBy: Sol-Moon Na; dateIdentified: 2025; **Event:** samplingProtocol: light traps; eventDate: 18-II-2017; **Record Level:** language: en; collectionCode: Insects; basisOfRecord: PreservedSpecimen
- **Type status:** Other material. **Occurrence:** recordedBy: Bae Y.S., Na S.M., Lee D.J., Ko J.H. Lee T.K., Cha Y.B., Jang C.M.; individualCount: 1; sex: female; lifeStage: adult; establishmentMeans: Native; occurrenceStatus: Present; occurrenceID: 6222B025-B171-5D20-A5E0-F04519BE1A57; **Taxon:** taxonID: Native; scientificName: Atteva sciodoxa; parentNameUsage: Attevidae; namePublishedIn: Meyrick, 1908 XXXIX—New Micro-Lepidoptera from India and Burma. Records of the Zoological Survey of India 2(4), 395–400.; phylum: Arthropoda; class: Insecta; order: Lepidoptera; family: Attevidae; genus: Atteva; specificEpithet: sciodoxa; taxonRank: species; scientificNameAuthorship: Meyrick, 1908; vernacularName: Tongkat Ali Tiger Moth; taxonomicStatus: accepted; **Location:** country: Laos; countryCode: LA; stateProvince: Bolikhamsai Prov.; locality: PKK Natl` Park; verbatimElevation: 290 m; verbatimCoordinates: 18°25'11.75"N 103°5'12.59"E; georeferenceProtocol: label; **Identification:** identifiedBy: Sol-Moon Na; dateIdentified: 2025; **Event:** samplingProtocol: light traps; eventDate: 3-IX-2018; **Record Level:** language: en; collectionCode: Insects; basisOfRecord: PreservedSpecimen
- **Type status:** Other material. **Occurrence:** recordedBy: Bae Y.S., Qi M.J., Lee D.J., Ko J.H., Lee T.K., Cha Y.B., Jang C.M.; individualCount: 1; sex: female; lifeStage: adult; establishmentMeans: Native; occurrenceStatus: Present; occurrenceID: 84C4889D-ED0E-517D-A495-A934A579F9AF; **Taxon:** taxonID: Native; scientificName: Atteva sciodoxa; parentNameUsage: Attevidae; namePublishedIn: Meyrick, 1908 XXXIX—New Micro-Lepidoptera from India and Burma. Records of the Zoological Survey of India 2(4), 395–400.; phylum: Arthropoda; class: Insecta; order: Lepidoptera; family: Attevidae; genus: Atteva; specificEpithet: sciodoxa; taxonRank: species; scientificNameAuthorship: Meyrick, 1908; vernacularName: Tongkat Ali Tiger Moth; taxonomicStatus: accepted; **Location:** country: Laos; countryCode: LA; stateProvince: Bolikhamsai Prov.; locality: PKK Natl` Park; verbatimElevation: 470 m; verbatimCoordinates: 18°27'23.76"N 103°5'5.15"E; georeferenceProtocol: label; **Identification:** identifiedBy: Sol-Moon Na; dateIdentified: 2025; **Event:** samplingProtocol: light traps; eventDate: 26-VII-2019; **Record Level:** language: en; collectionCode: Insects; basisOfRecord: PreservedSpecimen

#### Description

Adult (Fig. 1c, d). Wingspan 23–27 mm (n = 10). Vertex tuft with dark grey scales, frons with appressed white scales continuing to around compound eye. Antenna filiform, 3/5 length of the fore-wing, scape white, flagellum dark brown. Labial palpus slender, upcurved, pointed at tip, 1^st^ segment outer margin dark grey and inner margin white, 2^nd^ segment white and dark grey scales mixed, 3^rd^ segment outer margin white and inner margin dark grey. Patagium white, orange-brown transverse scales 1/4 posteriorly; tegula orange-brown, with white marking near wing base; mesoscutum orange-brown, with white streak at 2/3 posteriorly and concave at middle. Legs with coxa orange-brown outer margin, white inner margin, femur orange brown on outer margin, white on inner margin, tibia fuscous with white spot at base, 1^st^ and 2^nd^ segments of tarsus fuscous with white spot near base, other segments fuscous. fore-wing base colour orange-brown with 26–34 white dots, outer margin deep grey and fringe white. Hind-wing and fringe deep grey. Abdomen orange-brown.

Male genitalia (Fig. 2c, d): Uncus bifid, each process stout, over three times as long as socius; socius apically elongated triangular, with long hairs from base to basal 2/3, blunt apex; gnathal process strongly sclerotised, tongue-shaped, granulate apical half, as long as saccus. Valva elongated oval, convex from basal to 1/4, about 2 times as long as socius; sacculus lobate at base. Saccus short, about 1/4 length of valva. Aedeagus relatively slender, slightly longer than valva; with cornuti composed of four rows of spinules, apex to posterior 1/4.

Female genitalia (Fig. 3b): Papilla analis bifid, semi-oval with hairs. Apophyses posteriores 1.2 times as long as apophyses anteriores. Lamella postvaginalis shallow, setose, semi-elliptical lobes. Ostium rounded. Ductus bursae stout, 2 times as long as corpus bursae; antrum at posterior 1/5 of ductus bursae, slightly narrow to posterior. Corpus bursae ovate, deeply curved at base; signum narrow ovate plate, narrow to apex, with exterior denticules closely and inner denticules loosely arranged.

#### Diagnosis

This species is superficially similar to *A. fabriciella* Swederus, 1787, but it can be distinguished by the following characters: outline of fore-wing and hind-wing are deep grey; comb-like spines present on the socii and apex of uncus larger and more slenderer respectively; costa of valva is lack of protrusion; corpus bursae lacks conspicuous pleats; signum is smaller than that of *A. fabriciella* (Sohn and Wu 2013).

#### Distribution

Laos (present study), Malaysia (Borneo, Labuan & Penang Island), Myanmar.

#### Ecology

**Host plant**: *Eurycoma longifolia* Jack (Simaroubaceae) (Abood et al. 2008).

#### Notes

This species is well-known as a pest of the Tongkat Ali in Southeast Asia.

## Analysis

### DNA barcoding

**Figure 4.**
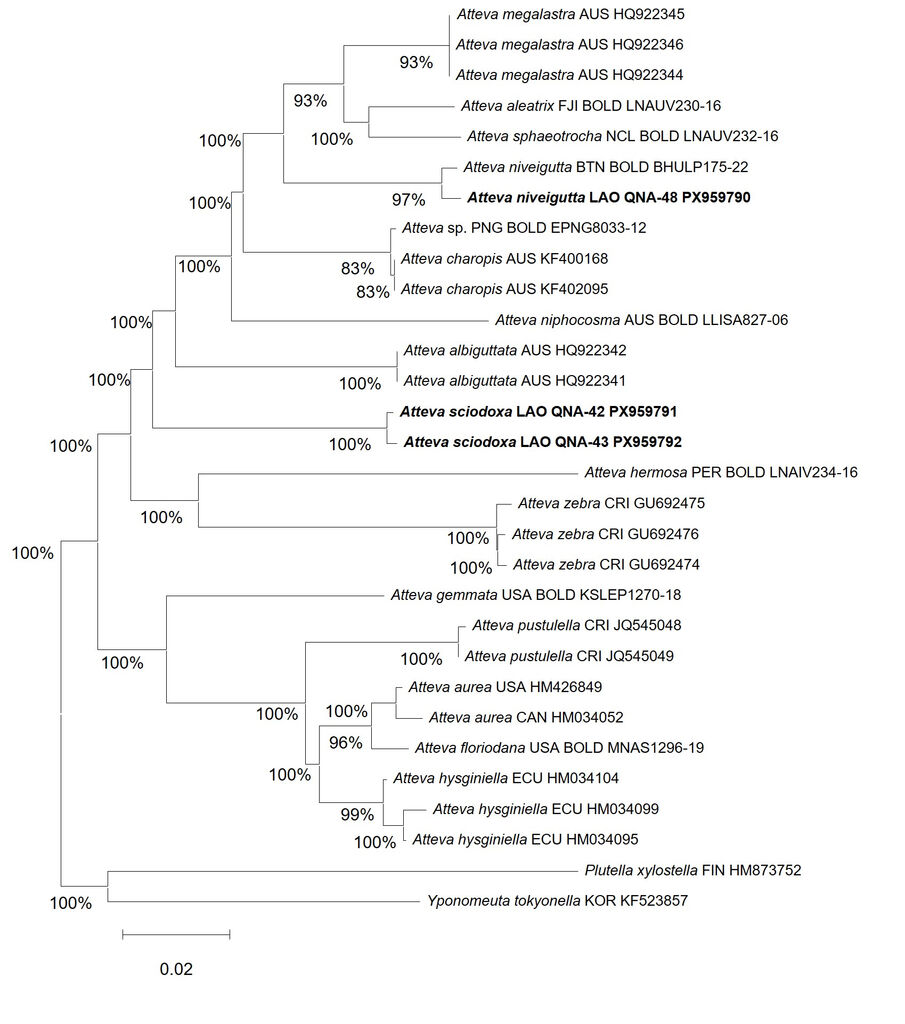
Neighbour-Joining (NJ) tree, based on COI barcode sequences (K2P distances) of *Atteva* spp. The analysis included 686 ingroup sequences of *Atteva* and two outgroup sequences, *Plutella xylostella* (HM873752) and *Yponomeuta tokyonella* (KF523857), for a total of 688 sequences. Bootstrap values (1,000 replicates) ≥ 70% are shown near nodes. Newly-generated sequences from Laos are indicated in bold. Scale bar = 0.02 substitutions per site.

The COI-based NJ tree recovered two well-supported clusters corresponding to the Laotian specimens. *Atteva niveigutta* sequence (GenBank PX959790) was grouped with other *A. niveigutta* sequences and the two Laotian *A. sciodoxa* sequences (GenBank PX959791–PX959792) formed a strongly supported clade (bootstrap 100%) distinct from congeners (Fig. 4). These barcode results are congruent with the morphology-based identifications and support the two species newly recorded from Laos.

## Supplementary Material

XML Treatment for Atteva

XML Treatment for Atteva niveigutta

XML Treatment for Atteva sciodoxa

## Figures and Tables

**Figure 1a.**
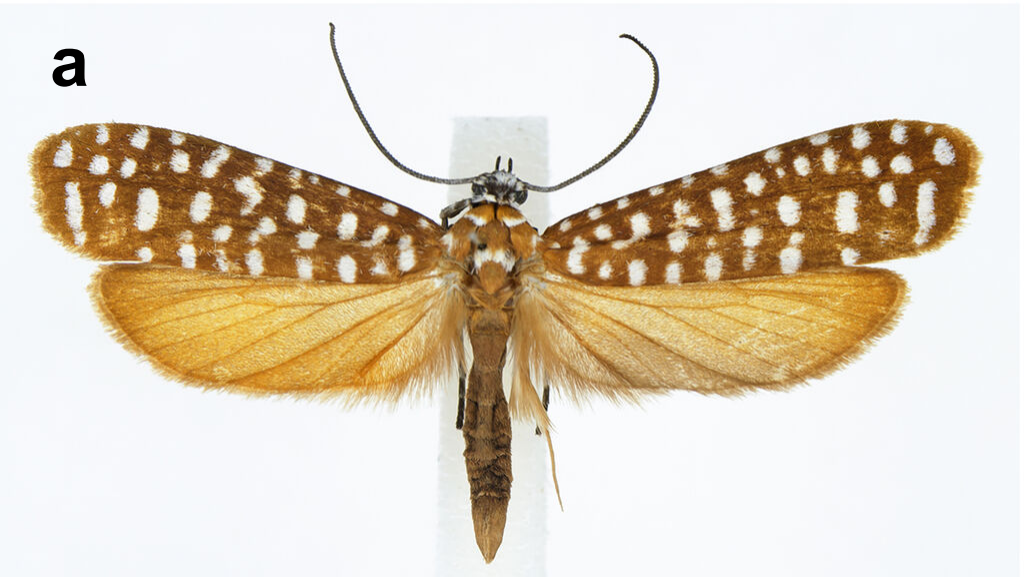
*A. niveigutta* Walker;

**Figure 1b.**
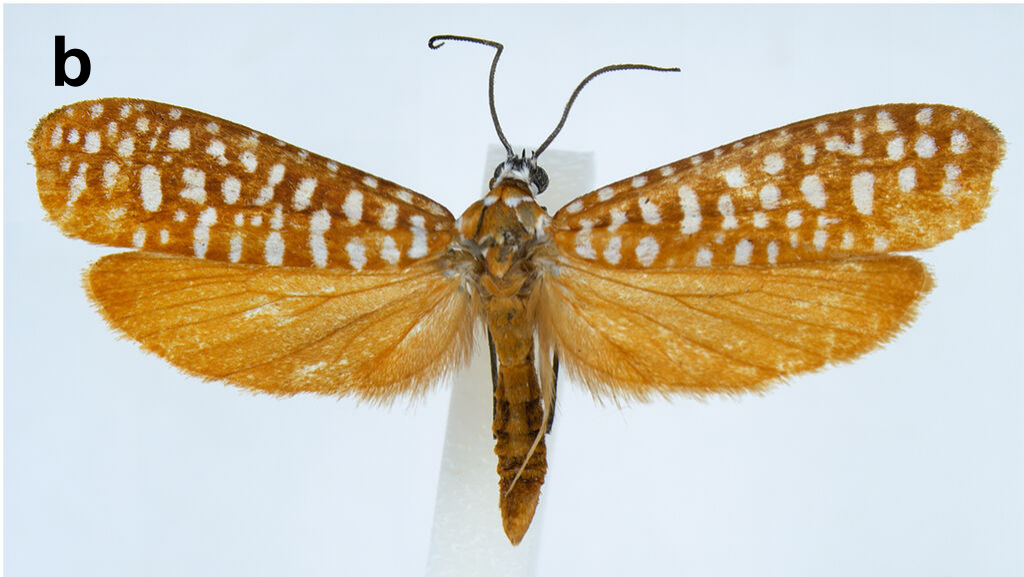
*A. niveigutta* Walker;

**Figure 1c.**
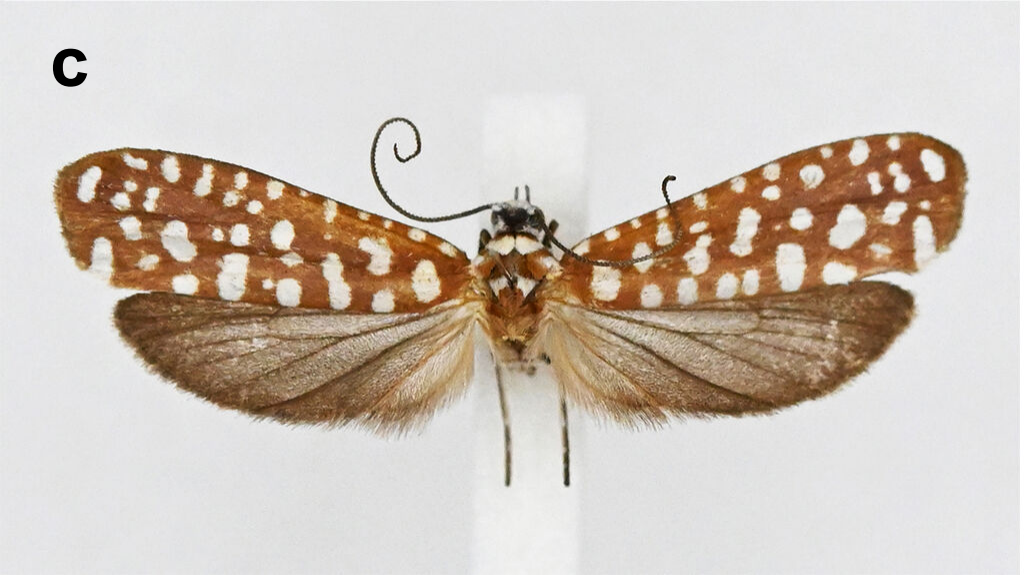
*A. sciodoxa* Meyrick;

**Figure 1d.**
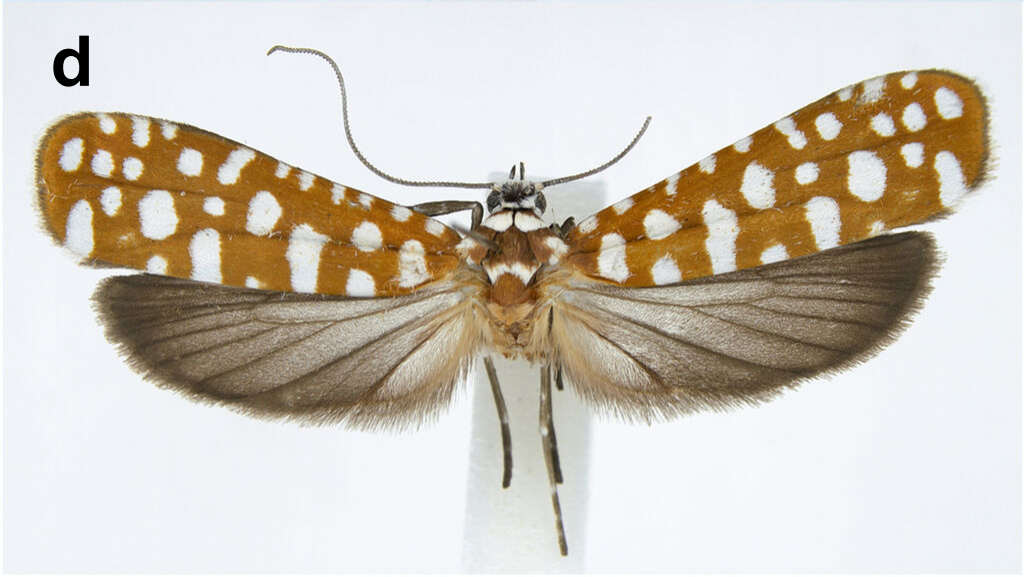
*A. sciodoxa* Meyrick.

**Figure 2a.**
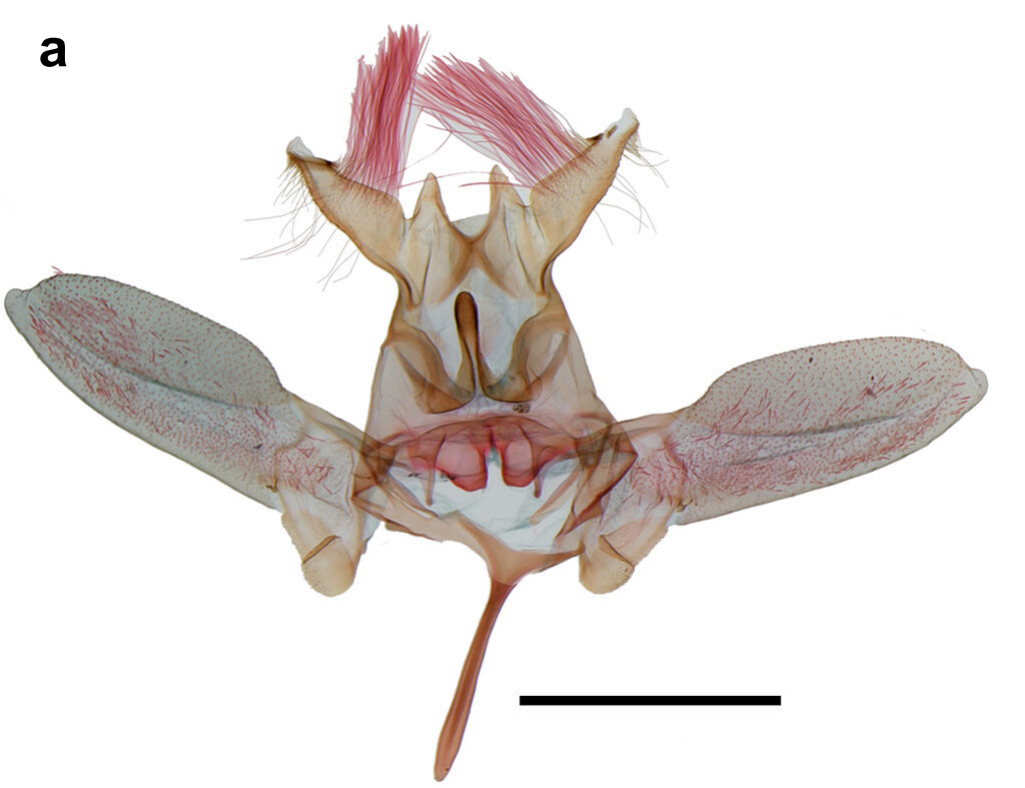
*A. niveigutta* Walker, genitalia;

**Figure 2b.**
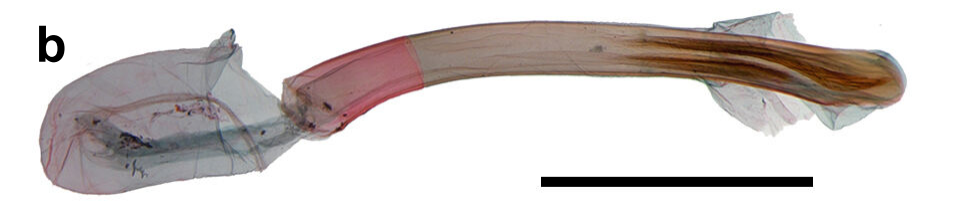
*A. niveigutta* Walker, aedeagus;

**Figure 2c.**
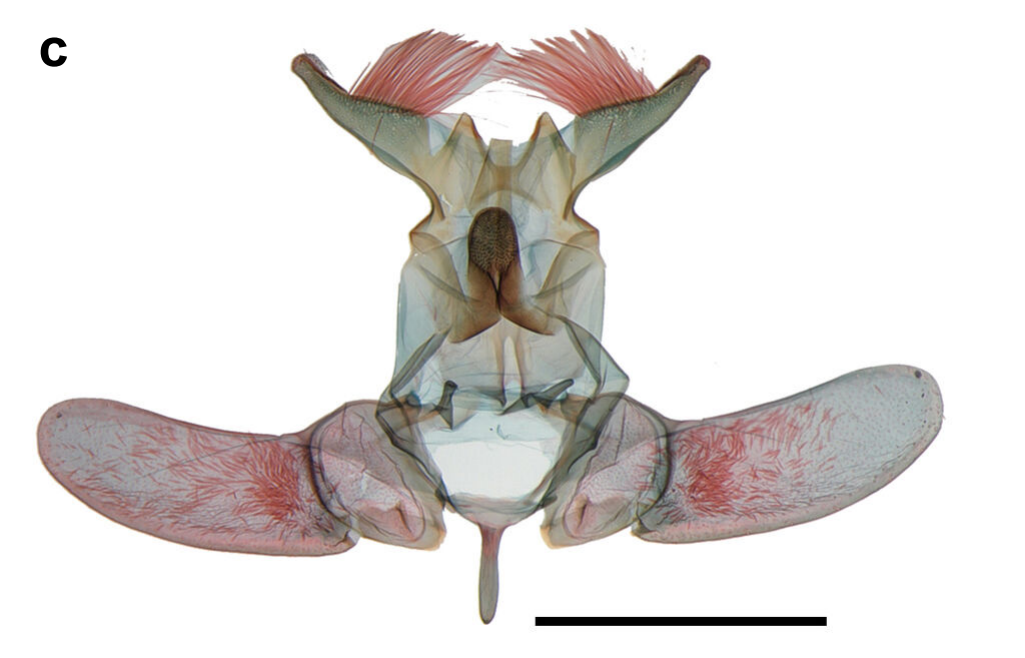
*A. sciodoxa* Meyrick, genitalia;

**Figure 2d.**
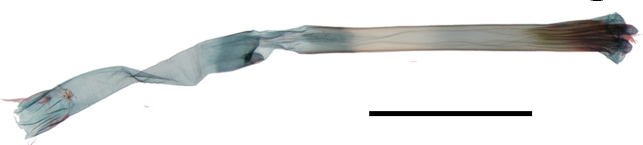
*A. sciodoxa* Meyrick, aedeagus.

**Figure 3a.**
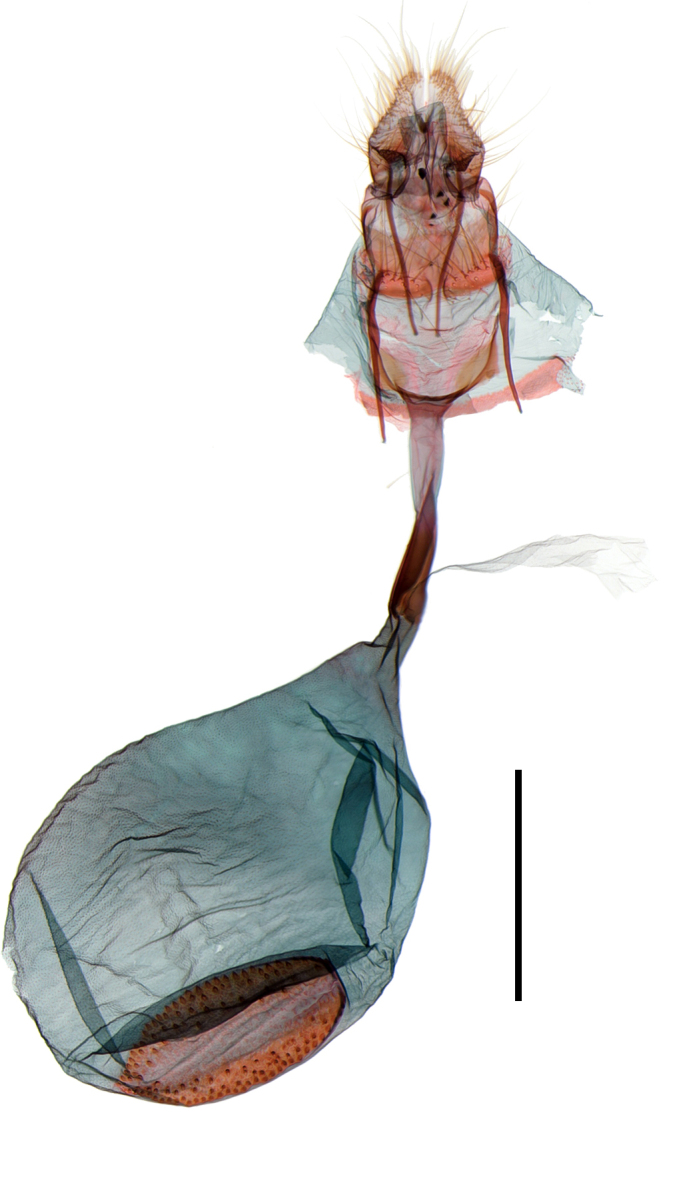
*A. niveigutta* Walker;

**Figure 3b.**
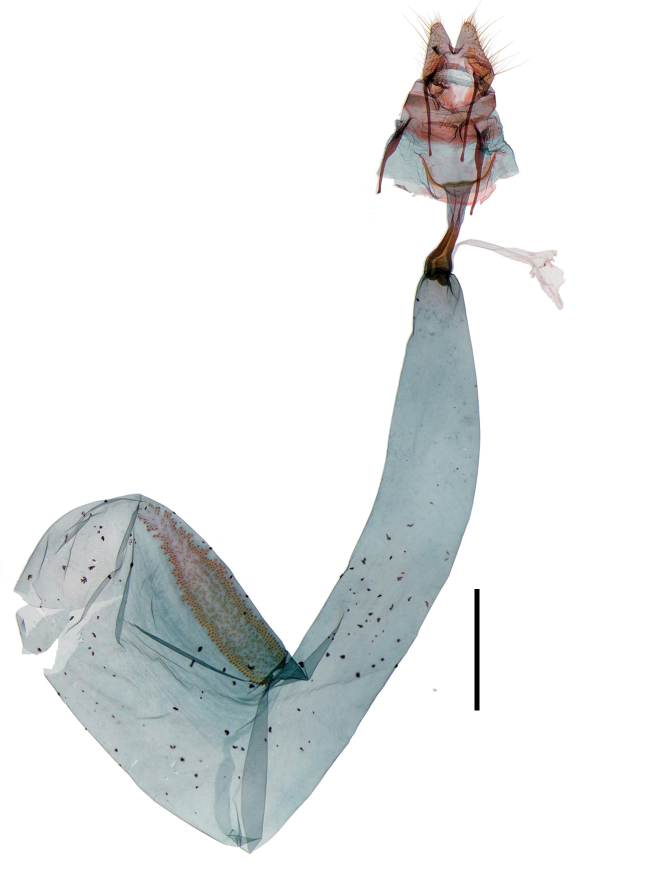
*A. sciodoxa* Meyrick.

**Table 1.** Representative COI sequences used in the Neighbour-Joining (NJ) analysis. Newly-generated sequences from Laos are indicated as “this study”. GenBank accessions and BOLD Process IDs are provided as given in the original records; voucher/specimen identifiers for reference sequences are not consistently available in public databases and are, therefore, omitted where unavailable.

**Taxon**	**Country**	**Voucher**	**Database**	**Accession no. /BOLD Process ID**	**Group**
*Atteva niveigutta*	LAO	QNA-48	GenBank	PX959790	ingroup (this study)
*Atteva sciodoxa*	LAO	QNA-42	GenBank	PX959791	ingroup (this study)
*Atteva sciodoxa*	LAO	QNA-43	GenBank	PX959792	ingroup (this study)
*Plutella xylostella*	FIN	—	GenBank	HM873752	outgroup
*Yponomeuta tokyonella*	KOR	—	GenBank	KF523857	outgroup
*Atteva albiguttata*	AUS	—	GenBank	HQ922342	ingroup
*Atteva albiguttata*	AUS	—	GenBank	HQ922341	ingroup
*Atteva aleatrix*	FJI	—	BOLD	LNAUV230-16	ingroup
*Atteva aurea*	USA	—	GenBank	HM426849	ingroup
*Atteva aurea*	CAN	—	GenBank	HM034052	ingroup
*Atteva charopis*	AUS	—	GenBank	KF400168	ingroup
*Atteva charopis*	AUS	—	GenBank	KF402095	ingroup
*Atteva floriodana*	USA	—	BOLD	MNAS1296-19	ingroup
*Atteva gemmata*	USA	—	BOLD	KSLEP1270-18	ingroup
*Atteva hermosa*	PER	—	BOLD	LNAIV234-16	ingroup
*Atteva hysginiella*	ECU	—	GenBank	HM034104	ingroup
*Atteva hysginiella*	ECU	—	GenBank	HM034099	ingroup
*Atteva hysginiella*	ECU	—	GenBank	HM034095	ingroup
*Atteva megalastra*	AUS	—	GenBank	HQ922344	ingroup
*Atteva megalastra*	AUS	—	GenBank	HQ922345	ingroup
*Atteva megalastra*	AUS	—	GenBank	HQ922346	ingroup
*Atteva niphocosma*	AUS	—	BOLD	LLISA827-06	ingroup
*Atteva niveigutta*	BTN	—	BOLD	BHULP175-22	ingroup
*Atteva pustulella*	CRI	—	GenBank	JQ545048	ingroup
*Atteva pustulella*	CRI	—	GenBank	JQ545049	ingroup
*Atteva* sp.	PNG	—	BOLD	EPNG8033-12	ingroup
*Atteva sphaeotrocha*	NCL	—	BOLD	LNAUV232-16	ingroup
*Atteva zebra*	CRI	—	GenBank	GU692476	ingroup
*Atteva zebra*	CRI	—	GenBank	GU692475	ingroup
*Atteva zebra*	CRI	—	GenBank	GU692474	ingroup
